# Supplementary material for: Mechanisms governing the pioneering and redistribution capabilities of the non-classical pioneer PU.1
Source: Nat Commun. 2020 Jan 21;11:402. doi: 10.1038/s41467-019-13960-2 (PMC6972792; doi:10.1038/s41467-019-13960-2)
Supplement: Supplementary file 7 — Source data [file 41467_2019_13960_MOESM7_ESM.zip › Source_Data/Figure6/Figure6B_MotifScanOutput/ETS1_mutPU.1/homerResults/motif8.similar.html]

motif8

## Information for motif8

G
T
A
C
G
A
T
C
C
A
G
T
A
G
T
C
A
T
G
C
G
A
T
C
G
T
A
C
A
G
T
C
C
T
G
A
A
T
G
C
G
T
C
A
  
Reverse Opposite:  

C
A
G
T
T
A
C
G
G
A
C
T
C
T
A
G
C
A
T
G
C
T
A
G
A
T
C
G
A
C
T
G
G
T
C
A
C
T
A
G
C
A
T
G
  

|  |  |
| --- | --- |
| p-value: | 1e-196 |
| log p-value: | -4.535e+02 |
| Information Content per bp: | 1.643 |
| Number of Target Sequences with motif | 3772.0 |
| Percentage of Target Sequences with motif | 27.97% |
| Number of Background Sequences with motif | 5505.4 |
| Percentage of Background Sequences with motif | 15.69% |
| Average Position of motif in Targets | 101.3 +/- 52.6bp |
| Average Position of motif in Background | 99.5 +/- 60.2bp |
| Strand Bias (log2 ratio + to - strand density) | 0.1 |
| Multiplicity (# of sites on avg that occur together) | 1.24 |
| Motif File: | file (matrix) reverse opposite |

### Similar de novo motifs found

|  |  |  |  |  |  |  |  |
| --- | --- | --- | --- | --- | --- | --- | --- |
| Rank | Match Score | Redundant Motif | P-value | log P-value | % of Targets | % of Background | Motif file |
| 1 | 0.888 | G A T C G A C T G A T C A G T C A G T C T G A C G A T C G T C A G T A C | 1e-157 | -362.990490 | 37.76% | 25.28% | motif file (matrix) |
| 2 | 0.765 | C A T G C A T G C T A G T A C G A C T G T A C G C T G A C T A G | 1e-156 | -360.652914 | 35.66% | 23.46% | motif file (matrix) |
| 3 | 0.954 | A T C G G A C T C A T G C A T G C T A G C A T G C T A G G T C A C T A G C A T G T C A G T C A G | 1e-150 | -347.599236 | 21.90% | 12.15% | motif file (matrix) |
| 4 | 0.946 | T C A G C A T G T A C G G C T A A C T G C A T G C T A G A C T G C T A G G T C A C T A G C A T G T C A G T C A G | 1e-142 | -328.726631 | 22.29% | 12.70% | motif file (matrix) |
| 5 | 0.903 | C T A G C T A G C T A G A C T G C A T G T C G A C A T G A C T G | 1e-118 | -273.287192 | 30.92% | 20.75% | motif file (matrix) |
| 6 | 0.874 | C G A T C A T G G A T C T C A G A C T G C T A G C T A G A C T G G T C A C A T G | 1e-67 | -155.546851 | 4.88% | 1.86% | motif file (matrix) |
